# Uncovering the Role of Tertiary Lymphoid Organs in the Inflammatory Landscape: A Novel Immunophenotype of Diabetic Foot Ulcers

**DOI:** 10.1111/jcmm.70479

**Published:** 2025-03-30

**Authors:** Deboshmita Banerjee, Shouvik Paul, Chitra Selvan, Sreekar Pai, B. S. Nandakumar, Souvik Mukherjee, Pongali B. Raghavendra

**Affiliations:** ^1^ National Institute of Biomedical Genomics Kalyani West Bengal India; ^2^ Regional Centre for Biotechnology (RCB) Faridabad Haryana India; ^3^ Department of Endocrinology and General Surgery M. S. Ramaiah Medical College and Hospitals Bengaluru India; ^4^ Department of Community Medicine M. S. Ramaiah Medical College and Hospitals Bengaluru India

**Keywords:** CD20^+^ B cells, CD3^+^ T cells, chronic vascular inflammation, diabetic foot ulcers, immune therapies, tertiary lymphoid organs (TLOs)

## Abstract

Diabetes foot ulcers (DFU) are the most common foot injuries leading to lower extremity amputation. Our study aimed to provide the first representative analysis highlighting the vital role of Tertiary Lymphoid Organs (TLO) inflammatory landscape in diabetic foot ulcers. The study explores mechanisms of TLO formation and the disease‐specific roles of TLOs in regulating peripheral inflammatory and immune responses. Additionally, comprehensive analysis of clinical data from DFU cases, focused on TLO pathophysiology and systemic immune‐inflammation landscape, is documented, aiming to identify the risk factors contributing to the development of DFUs. Our experimental results showed very significant differences were observed among the IL‐17 and IFN‐γ cytokine levels between the DFU vs. Control and DFU vs. NIDFU (Non‐Infectious Diabetic Foot Ulcers) groups, while minimal differences were observed in IL‐6 and TNF‐α cytokine levels. Immunohistochemistry staining or Immunophenotyping of DFU patient‐derived wound samples for TLO inflammatory stratification showed remarkable differences between DFU, NIDFU, and control groups both in CD3^+^ T Cells and CD20^+^ B cells. Overall, our study findings highlight the perspective role of TLO in DFU mechanisms and its prudent role in regulating peripheral inflammatory‐immune responses. TLO study‐related significant findings might be one of the important mechanisms, and its effective unveil might be a valuable treatment modality for DFU‐complications.

## 1 Introduction

Diabetic foot ulcers (DFUs) are a common debilitating complication affecting ~25% of individuals with both Type 1 and Type 2 diabetes mellitus (T1DM and T2DM) worldwide [1, 2]. DFUs are responsible for over 80% of non‐traumatic lower limb amputations and are associated with a high risk of recurrence and mortality [3]. Several risk factors contribute to the development of DFUs, including peripheral neuropathy [4, 5], peripheral artery disease [6], foot deformities [7], and poor glycemic control [8, 9, 10]. DFUs can lead to severe complications, such as infection, gangrene and eventually limb amputation, significantly affecting patients' quality of life [11]. Infections in DFUs are caused by a diverse range of pathogens, including Gram‐positive bacteria like *Staphylococcus aureus* and *Enterococcus spp*., Gram‐negative bacteria such as *Pseudomonas aeruginosa* and *Escherichia coli* , and anaerobes like *Bacteroides* spp [12, 13, 14, 15, 16, 17]. Recent clinical studies have reported an increasing prevalence of multidrug‐resistant (MDR) organisms, particularly methicillin‐resistant *S. aureus* (MRSA) and carbapenem‐resistant *Enterobacteriaceae*, complicating treatment outcomes [18, 19] Furthermore, studies highlight the role of biofilm‐forming pathogens in chronic DFUs, contributing to antibiotic resistance and impaired wound healing. Advances in metagenomic sequencing have revealed complex polymicrobial communities, including fungal co‐infections, influencing DFU progression and treatment efficacy. These findings underscore the need for targeted antimicrobial therapies and host‐directed strategies to combat persistent infections [20, 21, 22, 23, 24, 25, 26, 27]. One of the major factors contributing to the development and persistence of DFUs is chronic vascular inflammation [21, 22, 23], which plays a crucial role in disease progression and complications. Chronic vascular inflammation in DFUs is driven by a complex interplay between immune cells, endothelial cells, and extracellular matrix components [28]. Recently, a very handful of research has gained importance towards the tertiary lymphoid organs (TLOs) in regulating immune responses and maintaining chronic inflammation [29, 30, 31]. TLOs are ectopic, non‐encapsulated lymphoid structures that form in various inflamed tissues in response to local inflammation in conditions like autoimmune disorders, cancer, allogenic grafts or infection [32]. These structures resemble secondary lymphoid organs, such as lymph nodes and spleen, in their organisation and function but develop in response to local inflammation or infection. TLOs regulate local immune responses and perpetuate chronic inflammation [29, 33]. The formation of TLOs is a dynamic process orchestrated by a complex interplay between immune cells, stromal cells, and cytokines [34]. One of the key inducer cytokines produced by immune cells in the inflamed tissue, lymphotoxin‐α (LT α and lymphotoxin‐β) (LTβ), acts through the lymphotoxin‐β receptor (LTβR) or tumour necrosis factor receptor (TNFR) and contributes to the differentiation of stromal cells into lymphoid tissue organisers (LTo) [35, 36, 37]. These LTo cells further promote the organisation of TLOs by expressing homeostatic chemokines, including CCL19, CCL21, and CXCL13, which recruit additional immune cells to the site [38]. TLOs have been identified in the pancreas, adipose tissue, and vasculature in T1DM and T2DM, contributing to local inflammation and immune cell infiltration [39]. TLO formation has been linked to the severity of insulitis in T1DM. TLOs in the pancreas have been shown to contain CD4^+^ and CD8^+^ T‐cells and B‐cells, with B‐cells having a significant role in causing severe insulitis [40, 41]. The presence of immune cells in the adipose tissue of T2DM patients is associated with insulin resistance and systemic inflammation [42]. T helper (Th17 and Th1) cells in the TLOs can lead to germinal centre reactions supporting activation and differentiation of B cells that contribute to disease progression [43]. Fibroblasts and TLOs have been proposed to play a critical role in the pathophysiology of DFUs by modulating local immune responses, perpetuating inflammation, and promoting tissue damage [44]. The disease‐specific roles of TLOs in regulating peripheral immune responses in T2DM are an emerging area of research with potential implications for the development of targeted therapies.

CD3 (Cluster of differentiation 3) protein complex serves as a co‐receptor in mediating the activation of specific lineages of T lymphocytes (cytotoxic T cells and helper T cells) during adaptive immune response. Initially, CD3 is expressed in the cytosol of pro‐thymocytes but in the course of T lymphocyte maturation, its expression gradually increases in the cell membrane. CD3 and T cell receptor (TCR) functions synergistically to activate the T cells, where the specificity of binding is conferred by TCR and the signal transduction is provided by CD3 subunit [45].

CD20 (Cluster of differentiation 20) is a non‐glycosylated protein expressed on the surface of B cells and directly interacts with the B cell receptor (BCR). CD20 is expressed by the majority of the B cell subtypes (immature and mature) but not in terminally differentiated plasma cells [46, 47].

In this study, we have detailed the aetiology and pathophysiology of DFUs, the relation between microbial infections, DFUs, and chronic inflammation and various key serum‐based cytokine markers of DFUs. We categorised the study participants into three groups: healthy controls, diabetic foot ulcer (DFU) patients, and non‐infectious diabetic foot ulcer (NIDFU) patients. The healthy controls were individuals without diabetes or any history of foot ulcers. NIDFUs are a type of diabetic foot ulcer that lacks clinical signs of infection. Unlike infected DFUs, NIDFUs do not exhibit symptoms such as pus, increased warmth, redness, swelling, or foul Odour. Instead, these ulcers primarily arise due to neuropathy, ischaemia, or pressure‐related injuries rather than microbial invasion. This research study aims to provide an in‐depth analysis of the multifaceted role of formation, mechanisms of TLOs in the chronic vascular inflammation associated with DFUs while discussing their stratified numbers, including inflammatory or immune‐related mechanistic roles of TLO formation. Furthermore, the functions and intricate interplay between various immune cells in these microenvironments, their contribution to T1DM and T2DM, and potential therapeutic approaches of targeting TLOs for improving the treatment strategies or management and outcomes of patients suffering from DFUs‐related complications.

## 2 Materials and Methods

### 2.1 Clinical Subjects and Data

This study compared diabetic patients with foot ulcers (DFU) to those without (non‐DFU) at Ms. Ramaiah Hospital and IPGMER Hospital between 2021 and 2023. We categorised the study participants into three groups: 134 DFU patients, 50 NIDFU patients, and 50 healthy controls. DFU patients met the International Working Group on the Diabetic Foot criteria and had no history of amputation, gangrene, or severe ulcers (Wagner level 0‐III). Exclusions included T1DM, thyroid disorders, and acute diabetic complications. Data collected at admission included demographic information, diabetes duration, glycaemic control (HbA1C, glucose), peripheral neuropathy, vascular disease, foot deformities, and relevant lab tests. Non‐DFU patients and healthy controls were matched to DFU patients by diabetes duration and glucose control.

At admission, screening evaluated lower extremity circulation and nerve function, diagnosing PAD or DPN. Lab tests confirmed stable conditions, ruling out acute issues like myocardial infarction, stroke, and severe diabetic complications (e.g., hypoglycaemic coma, diabetic ketoacidosis, hyperosmolar hyperglycaemic state). Healthy controls (*n* = 50) were matched in a 3:1 ratio with DFU patients (*n* = 134) based on diabetes duration, glucose levels (fasting, postprandial), and HbA1C. T1DM, thyroid disorders, and extreme diabetic complications were exclusion criteria, with T2DM diagnosis following WHO guidelines. The characteristics and details of the DFU patients are enlisted and provided in the [Supporting Information S1].

### 2.2 TLO Stratification [Classification] Studies

Two locations of the foot (the heel and the first metatarsal) were examined, both of which have been reported to be locations with a high incidence of ulceration. Stereological methods and quantitative morphological techniques were used to evaluate the skin thickness, interdigitation index, elastic septate thickness and cell size. Histological structure and cell components of TLO were detected by immunofluorescence or immunohistochemistry staining for T cells zone (CD3 +) and B cells zone (CD20 +). TLO stage classification is stated as follows: Stage 0, normal tissue without TLO formation; Stage I, early TLOs with mixed T/B‐cell aggregates; Stage II, pre‐TLOs with segregated T/B‐cell area with lymph vessels and conduits; Stage III, well‐organised TLOs containing segregated T‐cell area and B‐cell follicles with germinal centres and follicle DC network.

### 2.3 Immunohistochemistry Staining

Immunohistochemistry was carried out on 5‐μm‐thick consecutive sections from paraffin‐embedded blocks using a slide processor. Briefly, the slides were incubated with primary antibodies for 1 h at room temperature. Antigen–antibody complexes were visualised using a peroxidase‐conjugated polymer backbone coupled to a secondary antibody system.

### 2.4 Cell Quantification Method

The slides were stained with anti‐CD3 and anti‐CD20 antibodies and were scanned with a high‐resolution scanner (NDP Slide scanner; Hamamatsu). Automatic cell counts of CD3^+^ and CD20^+^ cells were determined on 10 representative wound fields (4.4 mm^2^; original magnification 5×; 2560 × 2048 resolution) with Image J Software (NIH, Bethesda, MD). Furthermore, CD3^+^and CD20^+^ cells were counted semi‐quantitatively (score: 0, 1, 2, and 3 for none, low, intermediate, and high density of positive cells) to validate automatic cell count. We used the following cutoff points (highest tercile vs. 2 lowest terciles) to discriminate high and low densities of the different cell populations: CD3^+^ T cells, 270 cells/mm^2^; and CD20^+^ B cells, 135 cells/mm^2^.

### 2.5 Enzyme‐Linked Immunosorbent Assay (ELISA)

Cytokine measurements were performed according to the manufacturer's instructions (ELISA kits from eBiosciences). Briefly, 96‐well plates were sensitised with specific detection antibodies diluted in PBS and incubated overnight. After washing the plates with Tween 0.1% (v/v), the wells were blocked with BSA 1% (v/v) for 2 h. After further washing, 100 μL of samples, standards and blank were added to the plates and incubated overnight. After discarding the samples, detection antibodies were diluted in PBS and added to the plates. Another round of washing was performed, and streptavidin solution was added to the wells. The reaction was measured in a spectrophotometer at 490 nm.

### 2.6 DFU Wound Isolates Cultures and Microbiological Processing

One of the wound swabs was processed for aerobic bacteria by streaking on MacConkey agar. Individual colonies were processed by biochemical and microbiological techniques. These included the triple sugar iron test, the mannitol motility test, the urease test, the citrate test, the indole test, the hydrogen sulfide test, and the methyl red test for the identification of Gram‐negative bacteria and the coagulase test, the bile esculin agar test, and the catalase test for the identification of Gram‐positive bacteria. Catalase‐negative, coagulase‐positive bacteria were further tested by using the Kirby‐Bauer disk diffusion test with cefoxitin antibiotic. The MIC was determined according to CLSI regulations using HiComb MIC strips.

### 2.7 Ulcer Healing

As a surrogate endpoint for complete healing, we used the well‐established 50% wound size reduction after 4 weeks. Wounds that were ≥ 50% closed by week 4 were defined as “healing wounds”; all other wounds were defined as “non‐healing wounds” Ulcer size, including surface area and depth, was measured using planimetry. Ulcer size measurements were done pre‐ and post‐debridement weekly for the duration of the study.

### 2.8 Antibiotic Sensitivity Assays & Antibiotic Susceptibility Pattern

The antibiotic resistance pattern of bacteria was tested by disk agar diffusion (DAD) as recommended by the Clinical and Laboratory Standards Institute (CLSI). A disk containing antibiotics was used to determine the susceptibility of bacterial isolates according to the CLSI guidelines. *E. coli* ATCC 25922 and *S. aureus* ATCC 25923 were control strains. Isolates that showed resistance to at least one agent in three or more antimicrobial categories were considered.

### 2.9 Data Analysis and Statistics

In our current study, various statistical tests were utilised to analyse the clinical profiles, immune responses, and bacterial composition data in diabetic foot ulcer (DFU) patients. T‐tests and ANOVA were applied to compare continuous variables like ulcer duration and blood flow parameters among the patient groups, highlighting significant differences in disease progression and healing outcomes. An ANOVA test was used to assess differences in clinical parameters among groups of DFU patients. For cytokine analysis, pairwise t‐tests were conducted to compare cytokine levels between different groups, such as DFU, non‐infectious DFU (NIDFU), and control groups, highlighting significant differences in inflammatory responses. The chi‐square test was applied to evaluate the distribution of bacteria across different TLO grades and to determine the prevalence of antibiotic resistance patterns among bacterial isolates from DFU patient samples.

## 3 Results

### 3.1 Characterisation of DFU Patient Phenotype (Complete Clinical Profile)

The clinical profile of diabetic foot ulcer (DFU) patients highlights significant differences in disease progression, risk factors, and outcomes among groups with major amputation, non‐healing, and healing conditions. Non‐healing patients had a longer mean ulcer duration (34.3 ± 12.03 weeks, *p* = 0.046), indicating a chronic inflammatory state that may hinder healing. The major amputation group showed a higher prevalence of previous DFU history (58.87%) and more severe ulcers (Wagner grade 3.77 ± 1.34), suggesting a link between ulcer severity and the likelihood of amputation (*p* = 0.047). Although the ulcer area was larger in the major amputation group (37.65 ± 1.34 cm^2^), this was not statistically significant (*p* = 0.51). Healing patients had significantly higher ABI and TcPO2 values (*p* = 0.001 and *p* = 0.002, respectively), underscoring the importance of adequate blood flow and oxygenation for wound healing documented in the (Table 1). These findings emphasise the complexity of DFUs, where multiple factors interact to affect patient treatment modalities or prognosis.

**TABLE 1:** The DFU clinical profile key details and representative status of healing and non‐healing of DFU patients.

Different factors among DFU patients	Major amputation	Non‐healing (34)	Healing (100)	Total	*p*
Duration of DFU (mean [SD] weeks)	5.53 ± 3.12	34.3 ± 12.03**	15.23 ± 10.51*	17.65 ± 6.87	0.046
History of DFU (%)	58.87	50	27.18*	33.78	0.021
Wagner grade	3.77 ± 1.34	2.50 ± 1.04*	2.77 ± 0.90*	2.76 ± 0.97	0.047
Area of Ulcers (mean [SD] cm^2^)	37.65 ± 1.34	17.83 ± 5.54**	12.98 ± 8.94**	14.44 ± 10.16	0.51
Site of Foot Ulcers					
Fore (no.)	4	28	94		
Mid (no.)	2	8	44		
Hind (no.)	7	9	21		
ABI (mean [SD])	0.46 ± 0.27	0.76 ± 0.19*	0.82 ± 0.23**	0.97 ± 0.27	0.001
TcPO2 (mean [SD] mmHg)	31.85 ± 15.43	45.90 ± 19.54*	60.32 ± 20.17**	95.32 ± 35.89	0.002
*Gram*‐*Positive bacteria* (%)	56.42	65.93	23.45	60.43	0.073

*Note:* Major amputation compared with others.

* *p* < 0.05.

** *p* < 0.01; Non healing compared with healing.

### 3.2 Illustration of High‐Resolution DFU Images and the Details of Clinical Dataset

Types of chronic wounds observed in the studied patients, with a Venous leg ulcer, b pressure ulcer, c abscess ulcer, d surgical ulcer, and e others, are documented in the supplementary data information. The prevalence of DFU was found to be high in this cohort (95% CI: 18.98%–25.64%). Among different metabolic variants like hypertension, albuminuria, retinopathy, neuropathy, HbA1c, cholesterol, high density lipoprotein (HDL), low density lipoprotein (LDL) and triglyceride, only the duration of diabetes was significantly associated with DFU (*p* < 0.0018) as shown by logistic regression statistical analysis. Even after adjusting for all other potential risk factors, living with diabetes for more than 10 years is associated with an increase in the diabetic foot probability by 5.16‐folds (95% CI: 052–10.98 folds increase), *p* = 0.006. The adjusted effect for living with diabetes for more than 15 years on the diabetic foot complication probability is an increase by 1.53‐folds (95% CI: 0.39–4.37 folds increase), *p* = 0.005. However, living with diabetes for more than 5 years had a non‐significant adjusted effect on diabetic foot probability. Images of the DFU patients are provided in the (Figures S1 and S2).

### 3.3 TLO Classification Studies Based on Immune Cell Phenotype Profiling

**FIGURE 1:**
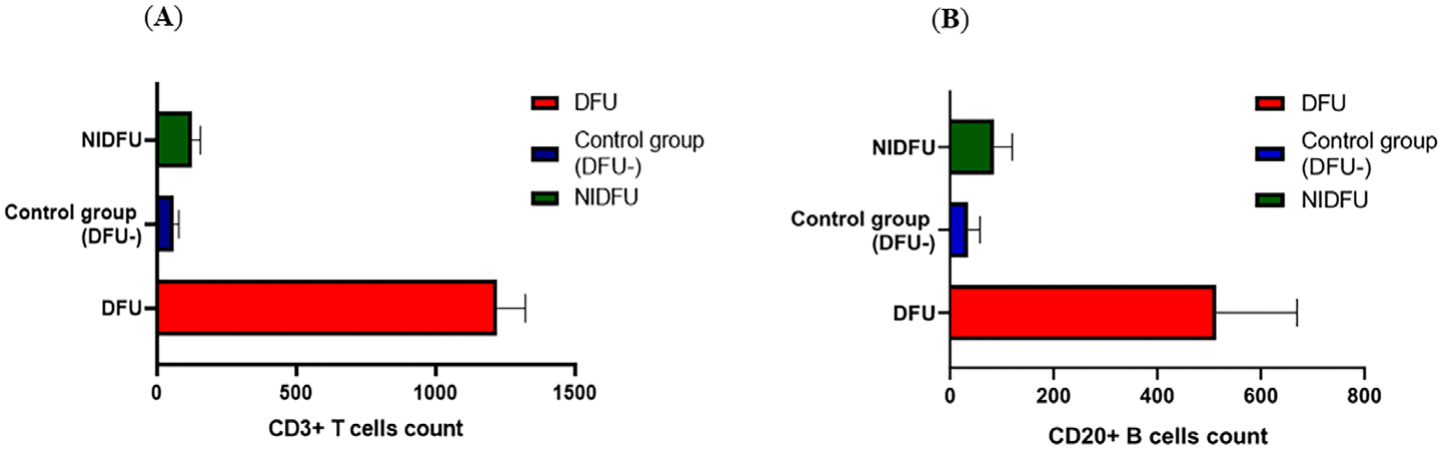
TLO stratification study by Immunophenotyping of wound samples in DFU, NIDFU and Control groups: (A) CD3^+^ T cells and (B) CD20^+^ B cells [DFU Vs NIDFU, *p < 0.05; DFU Vs Control, *p < 0.05; NIDFU Vs Control, *p value < 0.05]. Note : [Modifed Image attached with minor correction ‐ CD20+ B cells count].

The CD markers, CD3^+^ and CD20^+^, are very critical in understanding immune responses in DFU. CD3^+^ T cells represent the cellular component of the adaptive immune response and are crucial for orchestrating the immune defences against pathogens. CD20^+^ B cells are integral to humoral immunity, producing antibodies that target bacterial infections. Significant differences (*p* < 0.05) in both CD3^+^ T cells and CD20^+^ B cells among DFU, NIDFU, and control groups highlight the involvement of both humoral and adaptive immune responses in DFU, as shown in (Figure 1A,B). The presence of these immune cells within TLOs suggests that TLO formation in DFU may be a compensatory mechanism to bolster local immune responses against persistent infections and chronic inflammation. These data, drawn from 134 DFU patients, 50 NIDFU patients, and 40 controls, underscore the complex interplay of immune cells and cytokines in the pathophysiology of DFU, emphasising the role of TLOs in modulating these responses to influence patient outcomes.

### 3.4 Modulation of Cytokine Levels Mediated by TLOs in DFU Patient Sample Studies

The modulation of cytokine levels by tertiary lymphoid organs (TLOs) is pivotal in understanding the inflammatory responses in diabetic foot ulcer (DFU) patients. Cytokines such as IL‐17, IFN‐γ, IL‐6, and TNF‐α are critical mediators of inflammation and immune regulation. IL‐17 is involved in the recruitment of neutrophils and the promotion of inflammation, which can contribute to tissue damage if uncontrolled. IFN‐γ is a key player in promoting macrophage activation and enhancing antimicrobial responses, which are essential in controlling infection. IL‐6 acts as both a pro‐inflammatory cytokine and an anti‐inflammatory mediator, playing a role in the acute phase response and influencing wound healing. TNF‐α is a major pro‐inflammatory cytokine that can exacerbate tissue destruction and is associated with chronic inflammation seen in non‐healing wounds.

**FIGURE 2:**
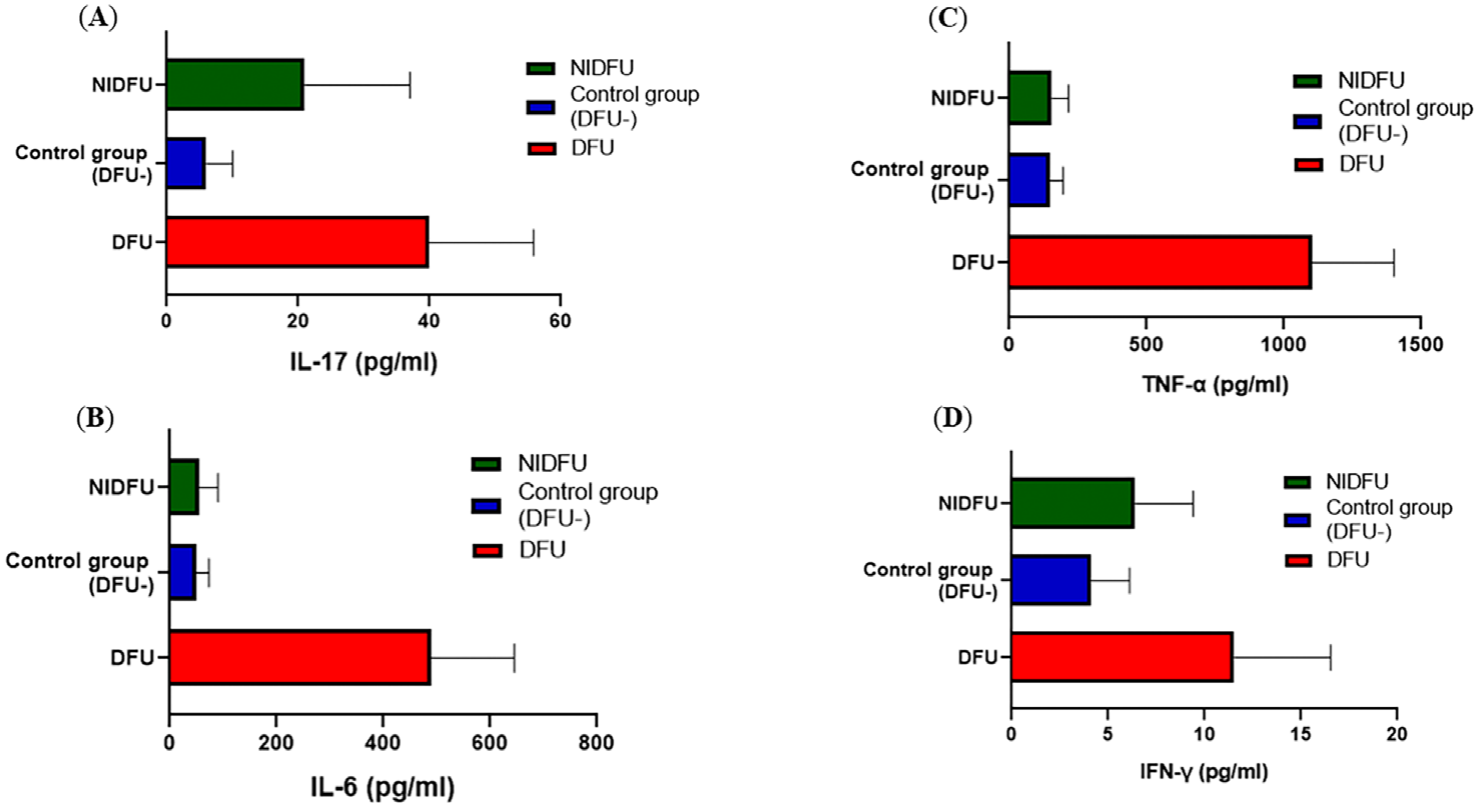
The levels of inflammatory cytokines investigated in DFU, NIDFU and Control groups: (A) IL‐17, (B) TNF‐α, (C) IL‐6 (D) IFN‐γ [DFU vs. NIDFU, *p < 0.05; DFU Vs Control, *p < 0.05; NIDFU Vs Control, *p value > 0.05].

Significant differences (*p* < 0.05) in IFN‐γ and IL‐17 levels were observed among DFU, non‐infectious DFU (NIDFU), and control groups, indicating heightened inflammatory responses in DFU patients. For IL‐6 and TNF‐α, significant differences (p < 0.05) between DFU vs. control and DFU vs. NIDFU groups suggest an elevated pro‐inflammatory state in DFU cases, which may contribute to poor healing outcomes, as represented in the data shown in (Figure 2A–D) However, insignificant differences were observed between control and NIDFU groups, which is indicative of a closer association of these cytokines with the infectious and inflammatory nature of DFUs.

### 3.5 Extraction and Quantification of Pre‐Dominant Bacterial Species From DFU Wound in a Clinical Cohort Study

The microbiological landscape of diabetic foot ulcers (DFUs) is represented by collecting the 134 patient samples using wound swabs and pus for culture‐based bacterial detection. The microbiological analysis of diabetic foot ulcer (DFU) patient samples revealed a diverse bacterial profile. *Staphylococcus aureus* was found in 30 cases, with a significant presence across different patient samples. *Streptococcus pyogenes* was identified in 15 cases, primarily among certain patient groups. Among the Gram‐negative bacteria, *Escherichia coli* showed a notable prevalence with 21 cases, followed by *Pseudomonas spp*. with 30 cases and *Klebsiella pneumoniae* with 14 cases, indicating their substantial role in DFU infections.

**FIGURE 3:**
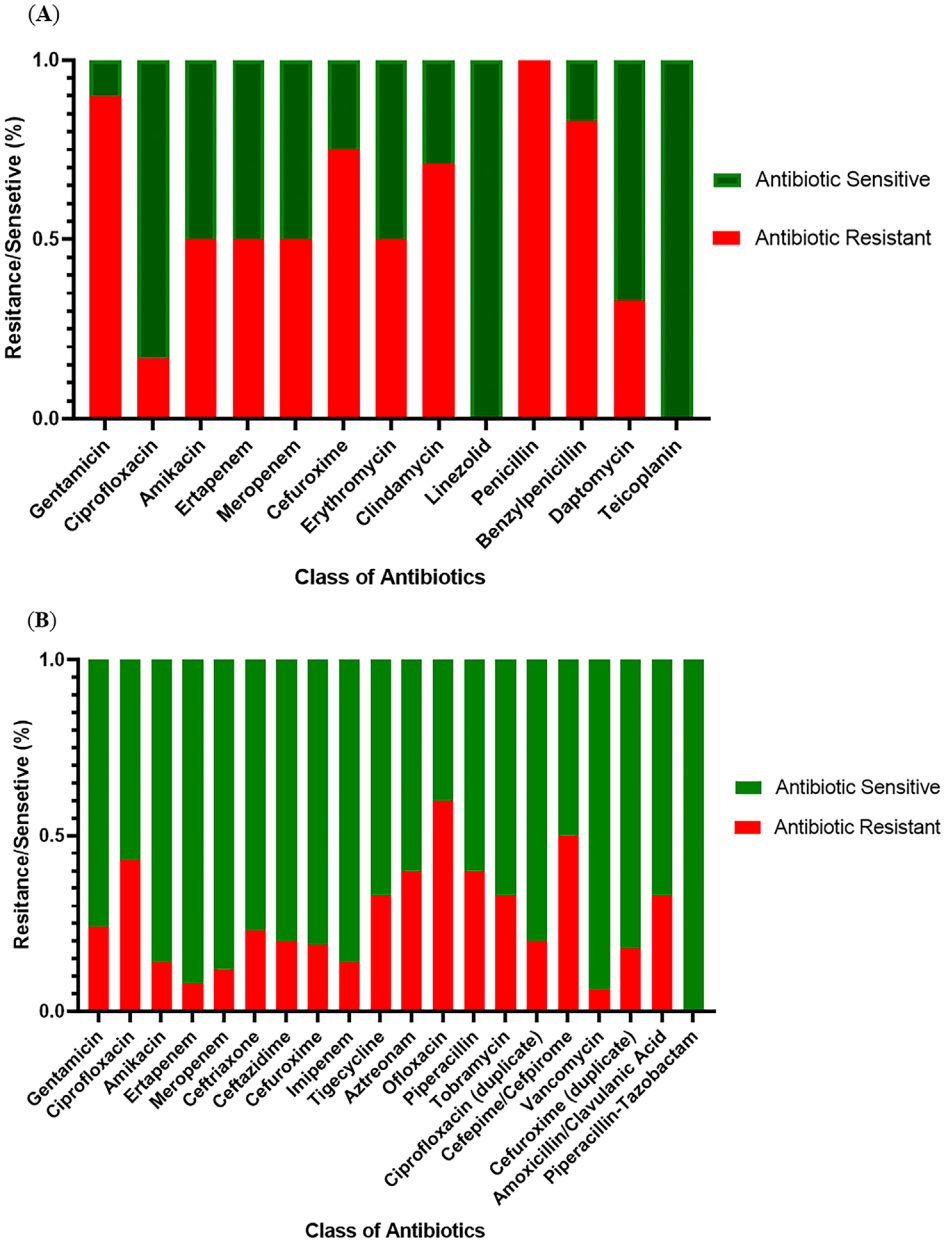
Microbiological study of DFU samples derived from clinical diseased subjects highlighting predominant bacterial species with variable antibiotic sensitivity/resistance pattern in the gram positive cocci and gram negative isolates, [*p value < 0.05]. as shown in (A, B).

*Citrobacter spp*. were found in 8 cases, while *Proteus mirabilis* was identified in 9 cases and *Proteus vulgaris* in 7 cases, highlighting their occasional but noteworthy presence. *Serratia* species were detected in 8 cases, whereas *Acinetobacter spp*. were present in 28 cases, underscoring their significant contribution to infections. Among Gram‐positive bacteria, *Enterococcus spp*. was isolated in 9 cases, and *Viridans streptococcus spp*. in 2 cases, showing their involvement in some DFU samples. *Anaerococcus* was also identified in 9 cases. Additionally, anaerobic bacteria such as *Porphyromonas* and *Actinomyces* were found in 6 and 8 cases, respectively, suggesting their role in chronic and mixed infections in DFUs. These findings highlight the complex and polymicrobial nature of infections in diabetic foot ulcers, emphasising the need for tailored antimicrobial therapy to effectively manage and treat these diverse bacterial populations, as shown in the data as (Figure 3A,B).

### 3.6 Bacterial Abundance in Different TLO

Study results documented the distinct distributions of gram‐positive and gram‐negative bacteria across different TLO grades in diabetic foot ulcers. Grade 1 TLOs mainly contained gram‐positive bacteria (60%), while Grade 2 had a majority of gram‐negative bacteria (70%). This trend continued in higher grades, with gram‐negative bacteria making up 80% in Grade 3, 75% in Grade 4, and 85% in Grade 5 TLOs (Figure S3). Pearson's Chi‐squared test showed a significant association between TLO grades and bacterial distribution (*χ* ^2^ = 35.017, df = 4, *p* < 0.001), highlighting variations in bacterial composition across TLO grades.

**FIGURE 4:**
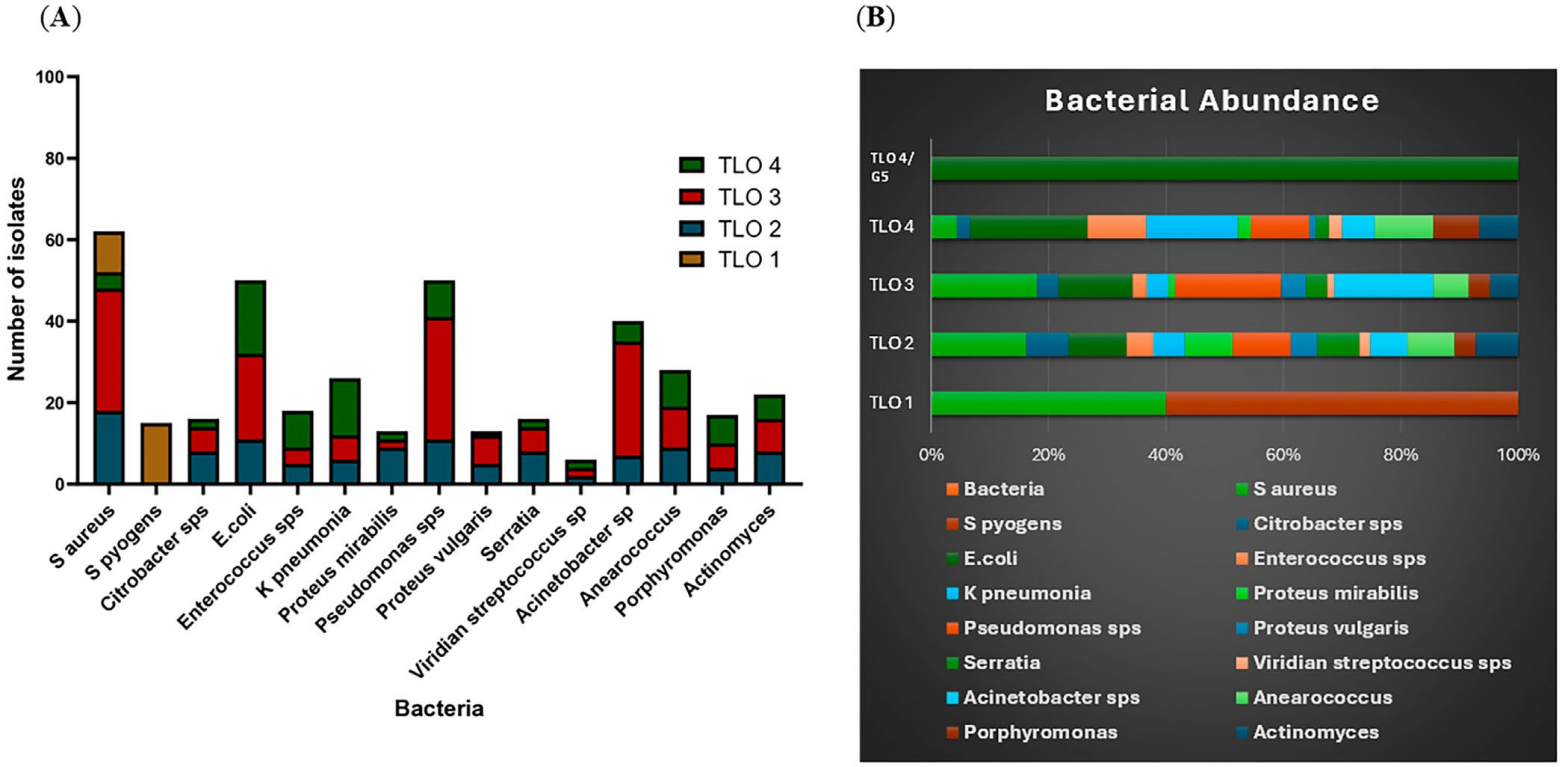
Bacterial Abundance in clinical isolates: (A) Distribution of predominant bacterial species during different grades of TLO in DFU wound samples; (B) Differential abundance of specific bacteria during the course of TLO formation, [*p value < 0.001].

These findings highlight the relationship between TLO grades and bacterial composition in diabetic foot ulcers, suggesting the need for further research into the mechanisms and therapeutic implications. Pearson's Chi‐squared test showed a significant association between TLO grades and bacterial distribution (*χ* ^2^ = 35.017, df = 4, p < 0.001), indicating significant variation in bacterial composition across TLO grades (Figure 4A,B). This indicates that the distribution of gram‐positive and gram‐negative bacteria varies significantly across different TLO grades in diabetic foot ulcers.

### 3.7 Antibiotic Resistance or Sensitivity Patterns in DFU Patient Sample Studies

Antibiotic sensitivity of DFU samples was tested using the Kirby‐Bauer method. Among *Staphylococcus aureus* isolates, 57% showed multidrug resistance (MDR), consistent with a study in Eastern India. Comparison of MDR‐MRSA cases between Eastern (9 cases) [48] and Southern India (4 cases) [30] showed no significant regional difference (chi‐square = 0.101, *p* = 0.75). Resistance was widespread among gram‐positive cocci to penicillin, amoxicillin, and gentamicin, while linezolid, teicoplanin, and ciprofloxacin were the most effective antibiotics.

**FIGURE 5:**
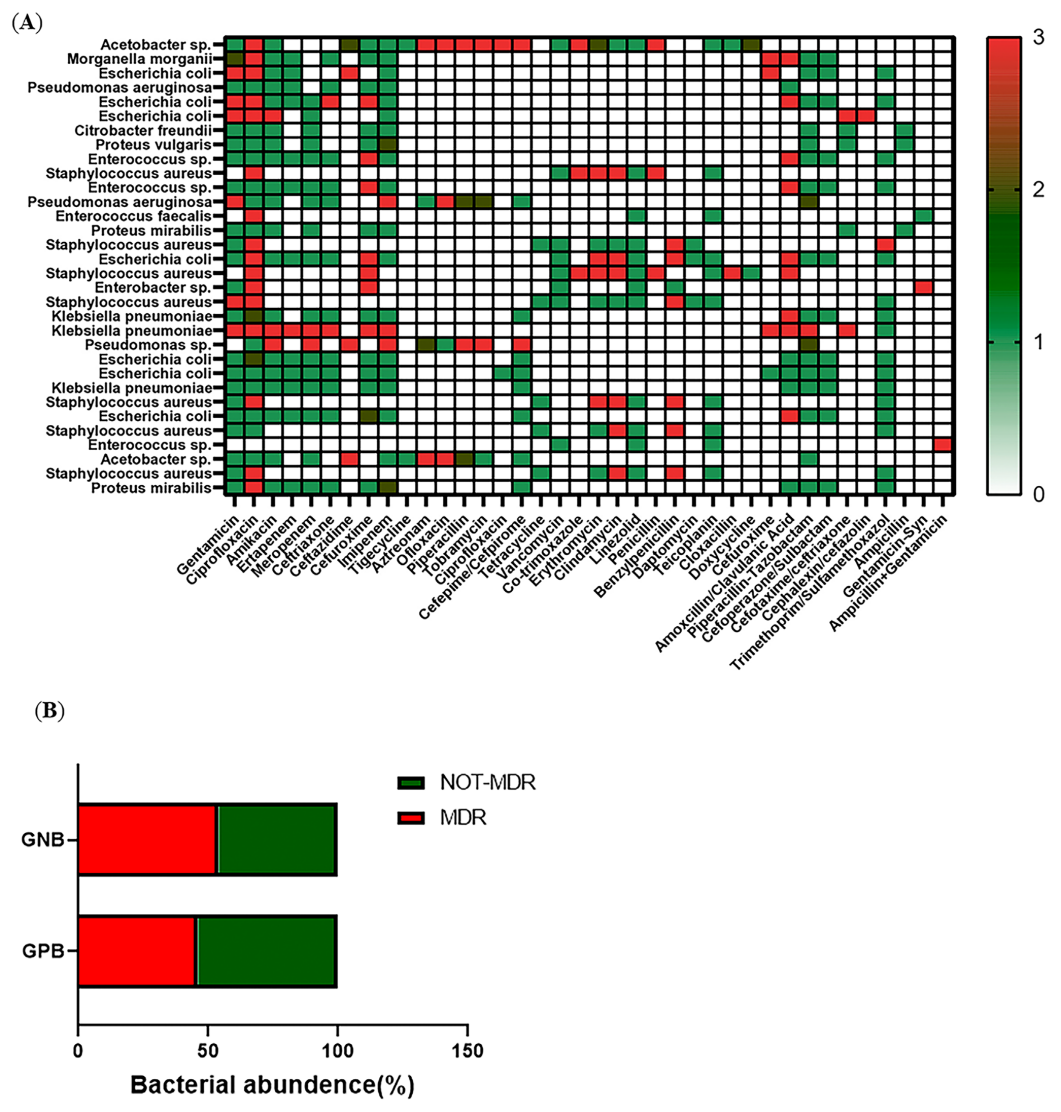
Antibiotic Susceptibility testing (A) Antibiotic sensitivity/resistance patterns of wound derived bacterial communities in DFU; (B) Proportion of multi‐drug‐resistant Gram‐positive and Gram‐negative bacteria in DFU wound samples, [*p value < 0.001].

Fifty‐seven percent of *E. coli* isolates showed multidrug resistance, higher than the 39% reported in a 2014 South India study [49]. Additionally, XDR and MDR *Proteus vulgaris* and *Proteus mirabilis* highlight concerns about anaerobic antibiotic resistance in diabetic foot ulcers (Figure 5A,B). Gram‐negative bacteria showed widespread resistance to ofloxacin, amoxicillin/clavulanic acid, and cefuroxime, while piperacillin‐tazobactam, trimethoprim, and imipenem were the most effective antibiotics, stressing the need to address these resistance patterns.

### 3.8 Overview of Epidemiological Status of DFU in Asia (India Region)

Diabetic foot ulcers (DFUs) affect 12%–15% of India's 77 million diabetic patients, with higher prevalence in rural areas due to delayed diagnosis and limited care. DFU infections are more common in males (71.59%) with an average age of 56.39 years. 85.01% of DFU patients tested positive for bacterial infections, with an average of 1.66 isolates per patient. Gram‐negative bacteria (64.06%) were more prevalent than Gram‐positive bacteria (36.51%), with *E. coli* and *S. aureus* being the most common isolates. Aerobic bacteria made up 85.6% of the isolates. Our findings align with other studies on DFU microbiology in India.

Consistent with other studies, *Staphylococcus aureus* was the most common Gram‐positive bacterium, found in 30 cases. Among Gram‐negative bacteria, *Escherichia coli* and *Pseudomonas spp*. were most prevalent, detected in 21 and 30 cases, respectively. This aligns with trends where Gram‐negative bacteria, especially *E. coli* and Pseudomonas, dominate DFU infections. Additionally, *Klebsiella pneumoniae* , *Acinetobacter spp*., and anaerobes like *Porphyromonas* and *Actinomyces* were identified, emphasising the complex, polymicrobial nature of DFUs. These findings highlight the need for tailored antimicrobial therapies to address the wide range of pathogens in DFUs.

## 4 Discussion

Our study presents a comprehensive characterisation of diabetic foot ulcer (DFU) patient phenotypes, delving into the intricate interplay of clinical, immunological, inflammatory, microbiological, and epidemiological factors that shape the disease progression and patient outcomes. The clinical profile of DFU patients indicates marked differences in disease progression, risk factors, and outcomes among groups classified into major amputation, non‐healing, and healing categories. Diabetic foot ulcers (DFUs) are severe complications in both type 1 diabetes mellitus (T1DM) and type 2 diabetes mellitus (T2DM), with significant clinical implications. Patients with T1DM often develop DFUs due to long‐term hyperglycemia‐induced neuropathy and microvascular dysfunction, whereas T2DM patients typically experience DFUs as part of a broader metabolic syndrome, including peripheral arterial disease, obesity, and chronic inflammation [23, 27]. The altered immune response in T1DM leads to impaired wound healing, while T2DM‐associated insulin resistance exacerbates inflammation and bacterial colonisation. Studies indicate that DFUs in T2DM are more prevalent, often linked to polymicrobial infections and biofilm formation, whereas T1DM‐related DFUs are associated with autoimmune‐driven microangiopathy [50]. Treatment for diabetic foot ulcers (DFU) is similar for both type 1 and type 2 diabetes but varies slightly due to different disease profiles. Both types require offloading, debridement, infection control, glycemic control, and patient education. However, type 1 diabetes often needs more intensive glycemic management and regular neuropathy assessments due to microvascular complications, while type 2 diabetes places more emphasis on vascular assessments like ABI testing, managing peripheral artery disease (PAD) with potential revascularisation, and addressing lifestyle factors such as smoking and weight management. A multidisciplinary approach involving podiatrists, endocrinologists, vascular surgeons, and wound care specialists, along with an individualised treatment plan based on ulcer severity and patient health, is essential for both types [23]. Patients with non‐healing ulcers in T2DM exhibited a significantly longer mean ulcer duration, averaging 34.3 ± 12.03 weeks, indicative of a prolonged inflammatory state that potentially impedes healing (*p* = 0.046). This chronic inflammation likely contributes to the persistent non‐healing status observed in these patients. In contrast, the major amputation group demonstrated a higher incidence of severe ulcers, with a mean Wagner grade of 3.77 ± 1.34, and a notable history of previous DFU occurrences (58.87%), underscoring a correlation between ulcer severity and the increased likelihood of amputation (*p* = 0.047). Though the major amputation group also presented with a larger ulcer area (37.65 ± 1.34 cm^2^), this difference did not reach statistical significance (*p* = 0.51). Meanwhile, patients in the healing group exhibited significantly higher ankle‐brachial index (ABI) and transcutaneous oxygen pressure (TcPO2) values (*p* = 0.001 and *p* = 0.002, respectively), highlighting the critical role of adequate blood flow and tissue oxygenation in promoting wound healing and recovery. This detailed clinical profiling elucidates the multifactorial nature of DFUs, where a myriad of elements including ulcer duration, severity, blood flow, and oxygenation interact intricately to influence patient prognosis and treatment outcomes.

Complementing the clinical data, the study's exploration of the immunological landscape of DFU patients sheds light on the role of tertiary lymphoid organs (TLOs) and their impact on cytokine modulation and immune cell profiling.

Tertiary lymphoid organs (TLOs) have emerged as critical players in the immune response to chronic vascular inflammation in diabetic foot ulcers (DFUs). Studies have highlighted the complex interactions between TLOs, immune cells, various cytokines, growth factors, and other molecules in the context of chronic vascular inflammation. Future directions include elucidating the molecular and cellular mechanisms underlying TLO formation and maintenance, investigating the role of TLOs in the initiation and progression of DFUs, developing therapeutic strategies to modulate TLOs and their associated immune responses, and investigating the potential of TLOs as biomarkers for DFU prognosis and treatment response. The identification of specific TLO‐associated factors may serve as valuable prognostic markers and provide a basis for the development of personalised treatment strategies for patients with DFUs. Future research should focus on deepening our understanding of the role of TLOs in the pathophysiology of chronic vascular inflammation in DFUs, as well as exploring their potential as prognostic and therapeutic targets.

TLOs, ectopic lymphoid structures formed in response to chronic inflammation, are implicated in the modulation of key cytokines such as *IL‐17, IFN‐γ, IL‐6* and *TNF‐α*, which are crucial mediators of inflammation and immune responses. *IL‐17* plays a pivotal role in inflammation and immune regulation. Earlier studies have shown that Indian subjects with T2DM, with or without complications, have higher values of *IL‐17* as compared to healthy controls. Also, diabetic neuropathy and diabetic retinopathy were positively correlated to levels of IL‐17. Further, levels of IL‐17 were positively correlated to hypertension and dyslipidemia, indicating a prevalent inflammatory state in the Indian population of T2DM with and without complications (*Journal of Diabetes Mellitus*>Vol.9 No. 4, November 2019). Our study findings also correlate that in DFU subjects, the cytokine levels of *IL‐17* are enhanced, and it increases inflammation, which alters the composition of the microbiome milieu to increase its pathogenicity.

Additionally, the study emphasises the relevance of immune cell profiling through CD markers such as CD3+ T cells and CD20+ B cells in understanding the immune dynamics in DFU. CD3+ T cells, representing the cellular component of the adaptive immune response, are crucial for orchestrating immune defences against pathogens. CD20+ B cells, integral to humoral immunity, produce antibodies targeting bacterial infections. The significant differences observed in both CD3+ T cells and CD20+ B cells across DFU, NIDFU, and control groups (*p* < 0.05) highlight the involvement of both cellular and humoral immune responses in DFU. The presence of these immune cells within TLOs suggests that TLO formation in DFU may serve as a compensatory mechanism to enhance local immune responses against persistent infections and chronic inflammation, thereby influencing patient outcomes. The significant alterations in CD3+ and CD20+ levels among different patient groups further reinforce the complexity of immune regulation in DFUs, suggesting that immune cell profiling could serve as a valuable tool for predicting disease progression and tailoring individualised therapeutic strategies.

The microbiological landscape of DFUs, as elucidated by this study, further adds to the complexity of managing this condition. The analysis of our study patient samples revealed a diverse bacterial profile, reflecting the polymicrobial nature of DFU infections. The findings underscore the prevalence of both Gram‐positive and Gram‐negative bacteria. This diverse bacterial presence indicates the need for tailored antimicrobial therapies that address the broad spectrum of pathogens typically found in DFUs. Additionally, the study explores the distribution of bacterial populations within TLOs of varying grades, revealing a dynamic shift from a predominance of Gram‐positive bacteria in Grade 1 TLOs (60%) to a dominance of Gram‐negative bacteria in higher‐grade TLOs (up to 85% in Grade 5). This trend underscores the evolving nature of bacterial communities within TLOs and their potential impact on local immune responses and inflammation, highlighting the necessity for further research to unravel the mechanisms driving these changes and their therapeutic implications.

Furthermore, the investigation into antibiotic resistance patterns among DFU patient samples reveals a concerning rise in multidrug‐resistant (MDR) and extensively drug‐resistant (XDR) bacterial strains. This escalating resistance trend poses a substantial challenge to the effective management of DFUs, necessitating the development of robust antimicrobial stewardship programmes and the exploration of novel therapeutic strategies.

This study provides a comprehensive analysis of DFU progression, emphasising the critical role of tertiary lymphoid organs (TLOs) in modulating peripheral inflammation and immune responses. Our findings reveal significant differences in IL‐17 and IFN‐γ cytokine levels between DFU, NIDFU, and control groups, while IL‐6 and TNF‐α levels show minimal variation. Immunophenotyping and immunohistochemistry analyses further demonstrate distinct TLO inflammatory stratifications based on CD3+ T cells and CD20+ B cells, highlighting their potential role in DFU pathology.

Given the increasing burden of multidrug‐resistant (MDR) infections, precise antimicrobial selection remains crucial. Current treatment regimens focus on targeted antibiotic therapy, biofilm‐targeting agents, and adjunctive interventions like negative pressure wound therapy (NPWT) and platelet‐rich plasma applications. Additionally, emerging immunomodulatory strategies, including IL‐17 or TNF‐α inhibitors, hold promise in mitigating chronic inflammation within DFU‐associated TLOs. Future research should explore TLO dynamics as potential prognostic markers and investigate microbiome‐based interventions, such as bacteriophage therapy or probiotics, to enhance DFU management. Advancing personalised medicine approaches that integrate immunological, microbiological, and clinical insights will be instrumental in improving therapeutic outcomes for DFU patients. In conclusion, this study provides a multi‐faceted examination of the factors influencing DFU progression and outcomes, highlighting the intricate interplay between clinical characteristics, immunological responses, microbiological profiles, and epidemiological factors. The insights gained from this research underscore the importance of a multidisciplinary approach to DFU management, integrating targeted antimicrobial therapies, immunomodulatory strategies, and comprehensive patient care to effectively address the diverse challenges posed by this debilitating condition.

## 5 Conclusion

Our study findings demonstrate the first representative analysis to show the vital role of Tertiary Lymphoid Organs inflammatory landscape in diabetic foot ulcers. The study findings provide insights and highlight the prospective role of TLO in DFU mechanisms and its prudent role in regulating peripheral inflammatory‐immune responses. TLO study‐related significant findings might be one of the important key mechanisms, and it's effective unveil might be a valuable treatment modality for DFU complications. Clinical profile of the DFU patients specifically validated for TLO indicates marked differences in the disease progression, risk factors, and outcomes among groups classified into major amputation, non‐healing, and healing categories. TLO study findings might also be one of key mechanisms and it's effective unveil might be a valuable treatment by shedding light on cytokine modulation and immune cell profiling modality by integrating the targeted antimicrobial therapies and immunomodulatory strategies for DFU complications.

## Author Contributions

**Deboshmita Banerjee:** data curation (lead), formal analysis (lead). **Shouvik Paul:** data curation (equal), formal analysis (equal). **Chitra Selvan:** data curation (equal), formal analysis (equal), investigation (equal), methodology (equal). **Sreekar Pai:** data curation (supporting), formal analysis (supporting), investigation (equal), methodology (equal). **B. S. Nandakumar:** methodology (supporting), project administration (supporting). **Souvik Mukherjee:** conceptualization (supporting), investigation (supporting), project administration (supporting). **Pongali B. Raghavendra:** conceptualization (lead), funding acquisition (lead), project administration (lead), writing – original draft (lead), writing – review and editing (lead).

## Ethics Statement

We adhered to the ethical and care guidelines of the Institute of NIBMG with approval ID as (NIBMG/2022/2/0032) and MSR hospitals with approval ID as (MSRMC/EC/AP‐02/07‐2022) and also were approved to ensure compliance.

## Conflicts of Interest

The authors declare no conflicts of interest.

## Supporting information


**Figure S1.** Wagner’s classification—Evaluation of DFU & Risk Stratification by representative illustration of High‐Resolution DFU Images from clinical subjects set of data −1 (A–D) and from clinical subjects set of data −2 (A–F).

**Figure S2.** Wagner’s classification—Evaluation of DFU & Risk Stratification by representative illustration of High‐Resolution DFU Images from clinical subjects set of data −1 (A–D) and from clinical subjects set of data −2 (A–F).

**Figure S3.** Enlisting the stages of TLO’s and representation of grading of inflammatory landscape of diabetic foot ulcers from clinical subjects derived data.

## Data Availability

The datasets generated during and/or analysed during the current study are available from the corresponding author upon reasonable request.
