# Supplementary figures and images for: Uncovering the Role of Tertiary Lymphoid Organs in the Inflammatory Landscape: A Novel Immunophenotype of Diabetic Foot Ulcers
Source: J Cell Mol Med. 2025 Mar 30;29(7):e70479. doi: 10.1111/jcmm.70479 (PMC11955414; doi:10.1111/jcmm.70479)

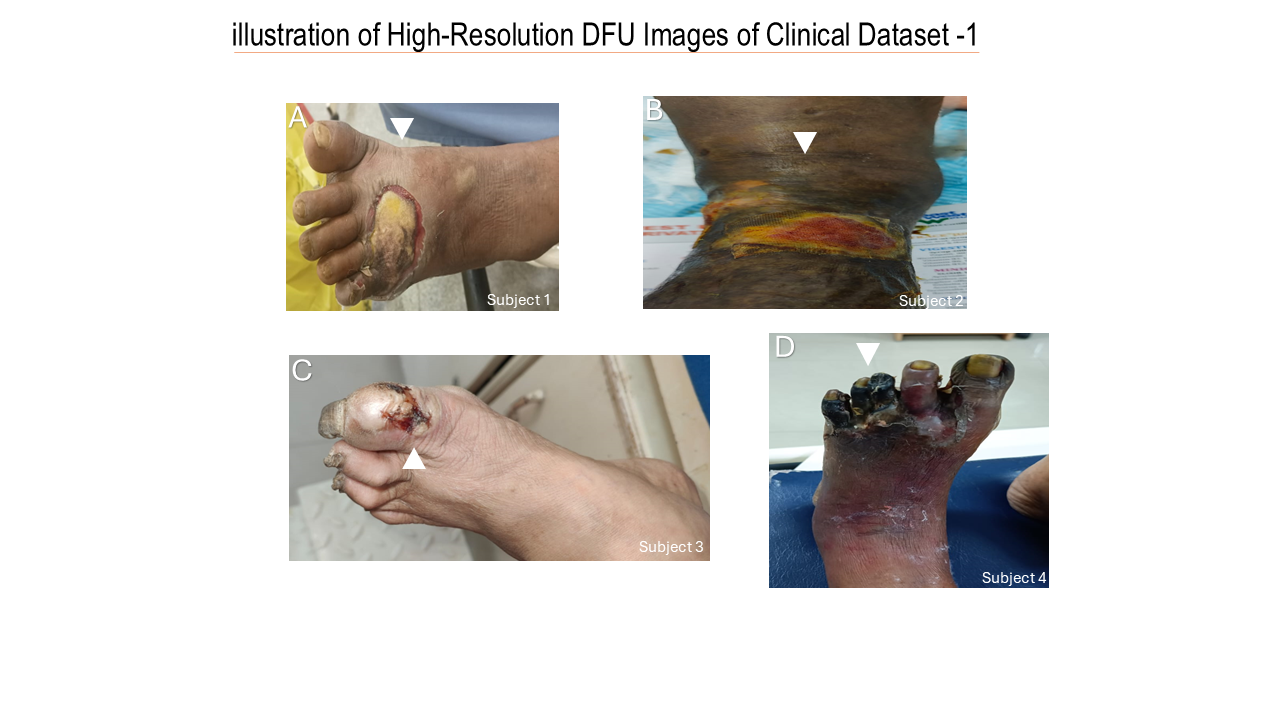

Supplement: Supplementary file 1 — Figure S1. Wagner’s classification—Evaluation of DFU & Risk Stratification by representative illustration of High‐Resolution DFU Images from clinical subjects set of data −1 (A–D) and from clinical subjects set of data −2 (A–F). [file JCMM-29-e70479-s001.tif]

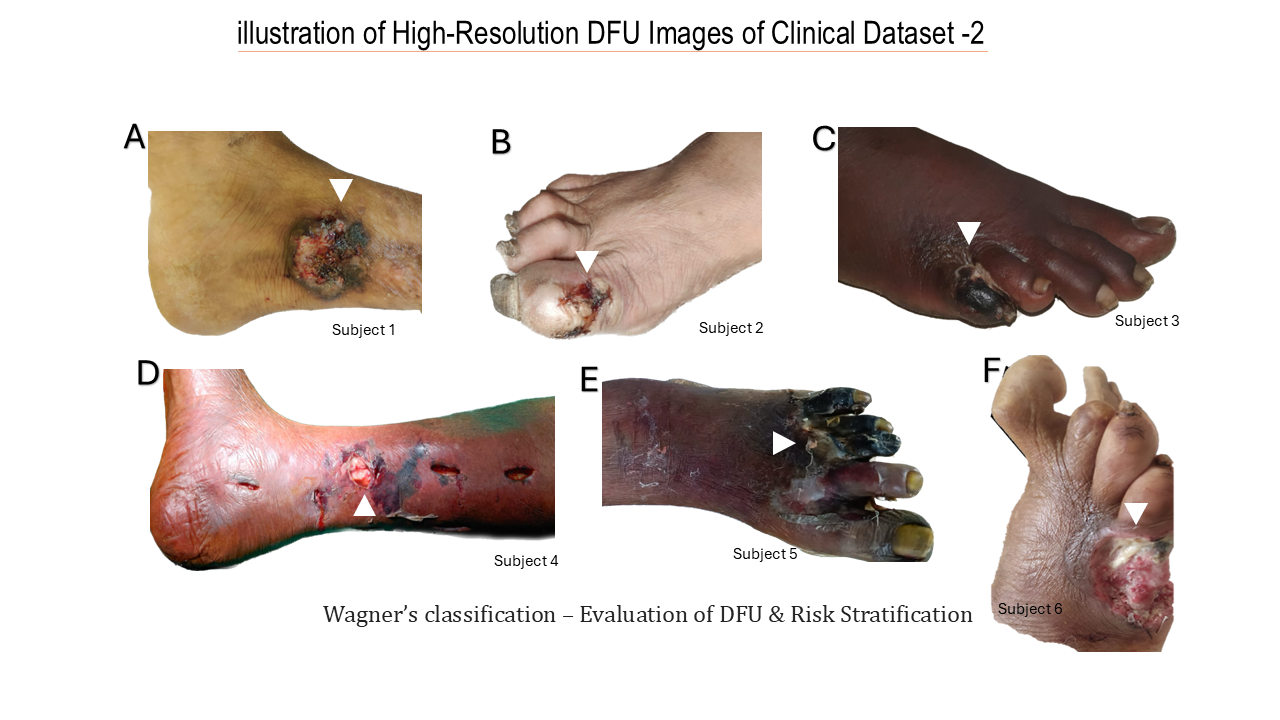

Supplement: Supplementary file 2 — Figure S2. Wagner’s classification—Evaluation of DFU & Risk Stratification by representative illustration of High‐Resolution DFU Images from clinical subjects set of data −1 (A–D) and from clinical subjects set of data −2 (A–F). [file JCMM-29-e70479-s002.tif]

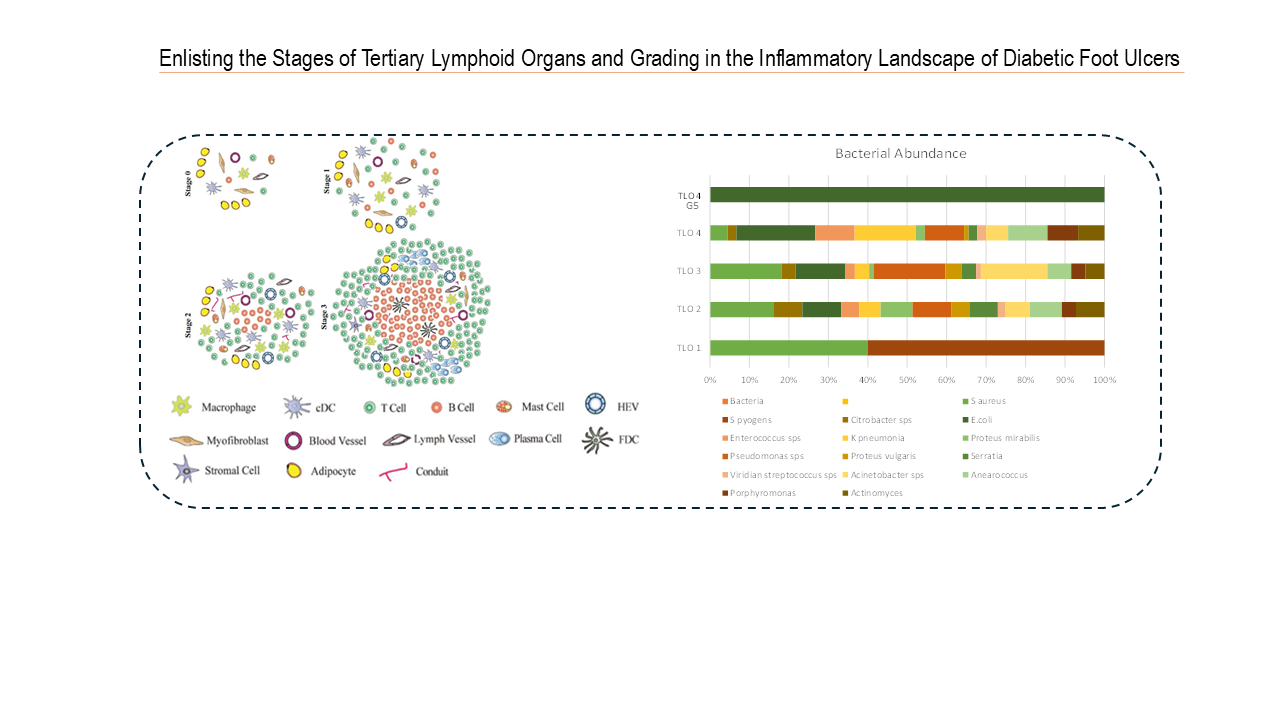

Supplement: Supplementary file 3 — Figure S3. Enlisting the stages of TLO’s and representation of grading of inflammatory landscape of diabetic foot ulcers from clinical subjects derived data. [file JCMM-29-e70479-s003.tif]
